# Funding and Innovation in Diseases of Neglected Populations: The Paradox of Cryptococcal Meningitis

**DOI:** 10.1371/journal.pntd.0004429

**Published:** 2016-03-10

**Authors:** Marcio L. Rodrigues

**Affiliations:** 1 Fundação Oswaldo Cruz (Fiocruz), Centro de Desenvolvimento Tecnológico em Saúde (CDTS), Rio de Janeiro, Brazil; 2 Instituto de Microbiologia Paulo de Góes, Universidade Federal do Rio de Janeiro (UFRJ), Rio de Janeiro, Brazil; University of California San Diego School of Medicine, UNITED STATES

## Introduction

It is estimated that fungal infections cause 1 million deaths annually, accounting for 50% of all AIDS-related deaths [1]. In this specific patient group, data from 2009 revealed that fungal meningitis is responsible for approximately 500,000 deaths each year [2].

The most common fungal pathogen infecting the brain is *Cryptococcus neoformans*, a yeast-like pathogen that is highly efficient in causing damage to immunosuppressed human hosts [3]. The fungus, which is widespread in the environment, primarily reaches the lung after inhalation of environmental cells and disseminates to the brain in the immunosuppressed host. Clinical studies in humans in combination with animal models of neurological cryptococcosis enabled the classification of the disease into different syndromes, including meningitis, encephalitis, meningoencephalitis, ventriculitis, increased intracranial pressure, and space-occupying lesions [3]. According to the Centers for Disease Control and Prevention (CDC) of the United States, cryptococcal meningitis is a global problem. Estimates by Park and colleagues indicated that approximately 957,900 cases (range, 371,700–1,544,000) of cryptococcal meningitis occur each year, resulting in 624,700 deaths (range, 125,000–1,124,900) by three months after infection [2]. Currently, estimates of global disease burden, including disability-adjusted life year (DALY), are not available, since cryptococcosis is not separately reported. Most cases are opportunistic infections that occur among people with HIV/AIDS.

Although the widespread availability of antiretroviral therapy (ART) in developed countries has helped reduce cryptococcal infections in these areas, it is still a major problem in developing countries where access to healthcare is limited. Throughout much of sub-Saharan Africa, for example, *Cryptococcus* is now the most common cause of adult meningitis, which may kill as many people each year as tuberculosis [2]. The high fatality rate, especially in developing countries, stems from late diagnosis, access only to the least effective treatments, and from drug resistance when treated.

## The Complexity of Treating Human Cryptococcosis

In the last 30 years, only one new class of antifungal drugs has been developed (echinocandins), with no efficacy against *C*. *neoformans* [4]. Lack of innovation in the field may result from different reasons, including an international trend of exaggerated focus on basic sciences [5] and, possibly, reduced funding. Common antifungals used for human cryptococcosis are toxic (amphotericin B), not active (echinocandins), or quickly develop resistance (azoles) [4]. Hence, the need for new, improved, and affordable antifungal treatments with greater safety and efficacy is clear [6]. The gold standard antifungal regimen for the treatment of cryptococcal meningoencephalitis is the combination of amphotericin B (a six-decade-old polyene) with 5-fluorocytosine (an antimetabolite antifungal agent) [7]. Amphotericin B has significant nephrotoxicity and requires costly intravenous administration [8], limiting its use in regions without strong medical infrastructure [9,10]. In fact, amphotericin B is not available in most of the African continent, according to the Global Action Fund for Fungal Infections [11]. 5-Flucytosine is not widely available outside of resource-rich areas, and it has potential hematologic toxicities that must be monitored closely during medication administration [12]. Fluconazole is often used as an alternative antifungal agent because of its lower cost and potential for oral administration. However, it is a decidedly inferior treatment option for this condition, associated with poorer outcomes and more frequent relapses [13]. In South Africa, more than 60% of people with culture-positive relapsed disease had fluconazole resistance. It is estimated that more than 30% of patients with cryptococcal meningitis will have microbiological and/or clinical failure (and presumably die), despite receiving standard therapy [13]. In summary, there are significant complexities in the management of cryptococcosis.

## The Imbalance between Funding Actions and Clinical Importance in Human Cryptococcosis

The Global Funding of Innovation for Neglected Diseases (G-FINDER) has recently reported on the 2014 global investment in research and development (R&D) of new products for neglected diseases, based on information provided by almost 200 organisations worldwide [14]. This report, which covered R&D funding for 35 neglected diseases, revealed a total investment of US$3,377,000,000. As in previous reports, malaria and tuberculosis were among the “top tier” diseases, combining 35.5% of the total investment in neglected diseases. Despite the alarming mortality rates, cryptococcosis received less than 0.5% of the global R&D funding [14].

**Fig 1:**
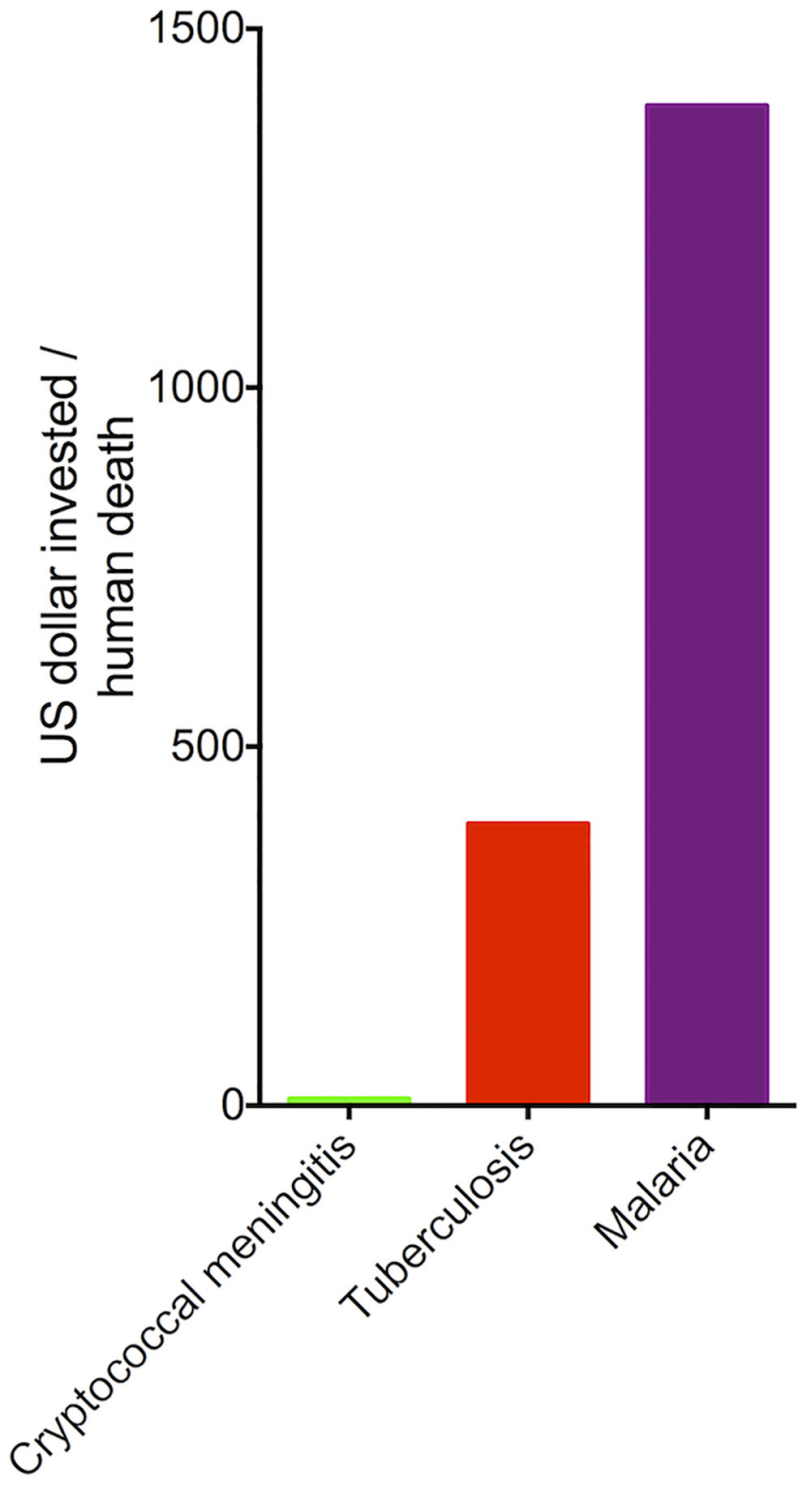
Funding and mortality ratios calculated for cryptococcosis, tuberculosis, and malaria. Values were obtained on the basis of median values of mortality rates provided by WHO (malaria and tuberculosis) and the United States CDC (cryptococcosis) and estimates of funding provided by the G-FINDER. Normalized values revealed a greatly reduced investment in cryptococcosis in comparison to tuberculosis and malaria.

The World Health Organization (WHO) estimated that 1.1 million (range: 0.97–1.3 million) people died in 2014 from primary tuberculosis, in addition to 0.39 million (range: 0.35–0.43 million) dying from HIV-associated tuberculosis [15]. According to the latest WHO estimates, released in September 2015, there were 214 million cases of malaria in 2015 and 438,000 deaths [16]. R&D funding in 2014 for tuberculosis and malaria corresponded to US$589 million and US$610 million, respectively [14]. Malaria mortality rates are similar to those estimated by the CDC for human cryptococcosis [2], which greatly contrasts with the US$5.8 million investment in R&D of this fungal disease [14], as summarized in Fig 1.

## Conclusions

Meningitis caused by *C*. *neoformans* kills more than 500,000 individuals per year worldwide. Estimates for R&D funding for human cryptococcosis in 2014 revealed indices of support that were greatly disproportionate to its importance for global health, in comparison to other neglected diseases. This scenario reveals an urgent need for innovation and increased support for R&D in the field of cryptococcal meningitis.

The importance of malaria, tuberculosis, and other diseases to global health is unquestionable, as is the need for investment in research, development, and innovation in these fields. However, the estimates calculated by the G-FINDER and summarized in this manuscript clearly indicate an imbalance between funding and mortality rates in the field of human cryptococcosis. This observation demonstrates that key action points are required to improve outcomes from cryptococcosis, the most fatal mycosis in AIDS patients.
